# Association of *NOS3* tag polymorphisms with hypoxic-ischemic encephalopathy

**DOI:** 10.3325/cmj.2011.52.396

**Published:** 2011-06

**Authors:** Radenka Kuzmanić Šamija, Dragan Primorac, Biserka Rešić, Bernarda Lozić, Vjekoslav Krželj, Maja Tomasović, Eugenio Stoini, Ljubo Šamanović, Benjamin Benzon, Marina Pehlić, Vesna Boraska, Tatijana Zemunik

**Affiliations:** 1 Department of Pediatrics, Clinical Hospital Centre Split, Split, Croatia; 2 University of Split, School of Medicine, Split, Croatia; 3 University of Osijek, School of Medicine, Osijek, Croatia; 4 Penn State University, Eberly College of Science, University Park, Pa, USA; 5 University of New Haven, West Haven, Conn, USA; 6 Department of Pediatrics, General Hospital Šibenik, Šibenik, Croatia; 7 Department of Medical Biology, University of Split, School of Medicine, Split, Croatia

## Abstract

**Aim:**

To test the association of *NOS3* gene with hypoxic-ischemic encephalopathy (HIE).

**Methods:**

The study included 110 unrelated term or preterm born children (69 boys and 41 girls) with HIE and 128 term and preterm born children (60 boys and 68 girls) without any neurological problems after the second year of life. Children with perinatal HIE fulfilled the diagnostic criteria for perinatal asphyxia. All children were admitted to the Clinical Hospital Split between 1992 and 2008. We analyzed 6 tagging single nucleotide polymorphisms (SNP) within *NOS3* gene (rs3918186, rs3918188, rs1800783, rs1808593, rs3918227, rs1799983), in addition to previously confirmed *NOS3*-associated SNP rs1800779. Genotyping was conducted using real-time polymerase chain reaction (PCR). Association analyses were performed according to allelic and genotypic distribution.

**Results:**

Allelic test did not show any SNP association with HIE. SNP rs1808593 showed genotype association (*P* = 0.008) and rs1800783-rs1800779 TG haplotype showed an association with HIE (*P* < 0.001). The study had 80% statistical power to detect (α = 0.05) an effect with odds ratio (OR) = 2.07 for rs3918186, OR = 1.69 for rs3918188, OR = 1.70 for rs1800783, OR = 1.80 for rs1808593, OR = 2.10 for rs3918227, OR = 1.68 for rs1800779, and OR = 1.76 for rs1799983, assuming an additive model.

**Conclusion:**

Despite the limited number of HIE patients, we observed genotypic and haplotype associations of *NOS3* polymorphisms with HIE.

Hypoxic-ischemic encephalopathy (HIE) in the perinatal period is a very serious neurological problem in children. It causes long-term or permanent damage, such as cerebral palsy, epilepsy, certain forms of mental retardation, and cognitive and sensory disorders in a relatively large number of affected children (1,2). HIE occurs in premature and full-term children after intrauterine infection and/or chorioamnionitis, premature rupture of membranes, maternal trauma, shock, or other etiologies. Premature infants are a particularly vulnerable group for the occurrence of hemorrhagic and hypoxic-ischemic brain damage, and the incidence of these injuries is higher in children with lower gestational ages (1,3). All of these conditions impair cerebral blood ﬂow, leading to ischemia and hypoxia and start a cascade of deleterious biochemical events that seriously and permanently injure the brain (2).

The main events during ischemia are an accumulation of free radicals, creation of excitatory neurotransmitters in selectively vulnerable areas, lack of adenosine triphosphate, and increase in intracellular calcium. Free radicals are highly reactive and cause damage of lipids, DNA, and proteins, which leads to neuronal death. One of the important radicals in this event is nitric oxide (2,4). Nitric oxide is a weak free radical formed during the conversion of l-arginine to L-citrulline by nitric oxide synthase (NOS). There are two isoforms of NOS: constitutive and inducible (NOS2). Constitutive NOS is active in vascular endothelial cells and is called endothelial nitric oxide synthase (NOS3), while the NOS which is active in the central and peripheral nervous system is called neuronal nitric oxide synthase (NOS1) (5).

The role of nitric oxide in the pathogenesis of ischemic brain damage is dual: protective and deleterious, depending on NOS isoform and the type of the cell that produces nitric oxide. It also depends on the term and intensity of oxidative stress (4). In an intense oxidative stress, nitric oxide produced by NOS1 leads to neuronal death, causing mitochondrial damage, loss of energy, and further disruption of calcium homeostasis (4). Nitric oxide produced by NOS3 has a protective role. Mild exposure to ischemia activates enzyme NOS3 and produces small amounts of nitric oxide with subsequent relaxation of blood vessels and vasodilatation. The nitric oxide produced by NOS3 plays a prominent role in maintaining cerebral blood flow and preventing neuronal injury (6). It has several different roles, such as inhibition of platelet aggregation, inhibition of platelet and leukocyte adhesion to endothelium, relaxation and inhibition of smooth blood vessel muscle cells proliferation, stimulation of angiogenesis, anti-inflammatory action, and prevention of oxidative damage (6). Because of the very important role of nitric oxide in these processes, impaired nitric oxide synthesis by NOS3 in vascular endothelial cells, may contribute to ischemic damage (7). Understanding this dual role of NOS and nitric oxide in the brain ischemia may lead us to prevention and treatment of ischemic damage (8).

Nitric oxide production in blood vessels is controlled by *NOS3* gene located on the chromosome 7q36.1, which contains 26 coding exons and has genomic size of 21kb (6). It has been reported that *NOS3* gene polymorphisms may lead to reduction or disruption of gene transcription, altering enzymatic function, and therefore cause susceptibility to ischemic brain diseases (9). So far, several gene polymorphisms of *NOS3* have been found to be associated with cerebrovascular disease: Glu298Asp, also known as E298D, or G894T (rs1799983) in exon 7; a variable number of 27 bp nucleotide tandem repeat sequences (VNTR, 27pb) in intron 4; and T786C polymorphism (rs2070744) in the 5′ region of *NOS3* (10). Another *NOS3* gene polymorphism, -922A/G (rs1800779), showed an association with ischemic stroke (11). However, the results are still ambiguous, and functional consequences of *NOS3* polymorphisms have not yet been completely clarified. There seems to be a correlation of *NOS3* polymorphisms with the clinical manifestation of perinatal and neonatal diseases (12). Our study aims to clarify the role and impact of 7 *NOS3* gene polymorphisms on the occurrence of perinatal HIE.

## Material and methods

### Participants

The study included 110 unrelated full-term or preterm born children (69 boys and 41 girls) with hypoxic-ischemic injury and intracranial hemorrhage (HIE group). The control group consisted of 128 apparently healthy full-term and preterm born children (60 boys and 68 girls), without any neurological problems after the second year of life (control group), who were admitted to the Clinical Hospital Split between 1992 and 2008. All children with perinatal hypoxic-ischemic injury fulfilled the diagnostic criteria for perinatal asphyxia: fetal heart frequency during delivery <100 or >160 per min, and/or Apgar score at 5 minutes ≤7, and/or blood gases pH≤7.1. The clinical course of HIE was mild, moderate, or severe for all the affected children according to the classification (13). The HIE was confirmed with brain ultrasound. Routine cranial sonography was performed on preterm infants (gestational age less than 37 weeks) on the first day of life and was repeated at least once during the first week and at the 2nd and at 4th-6th week of age. The cranial sonography was performed on infants born at 37 weeks gestational age or later depending on clinical indications. Hypoxic-ischemic injury and intracranial hemorrhage were confirmed by magnetic resonance imaging at the age of 2 years. The HIE group consisted of 72.7% of patients with hypoxic-ischemic injury and of 27.3% of patients with hypoxic-ischemic injury accompanied with intracranial hemorrhages. The study was approved by the Ethics Committee of Clinical Hospital Center Split and School of Medicine Split, and informed consent was obtained from patients’ parents prior to the blood sampling.

### DNA extraction and genotyping

Genomic DNA was extracted from peripheral blood leukocytes using the Perfect gDNA kit (Eppendorf, Hamburg, Germany). Six haplotype-tagging SNPs (rs3918186, rs3918188, rs1800783, rs1808593, rs3918227, rs1799983) were selected across the *NOS3* gene using the Tagger software (*http://www.broad.mit.edu/mpg/tagger/server.html*) (14). The proportion of variation across the *NOS3* gene region captured by tagSNPs was calculated based on the HapMap phase II using the same software (15). The SNP rs1800779 was also analyzed in this study because of its more common association with the development of HIE (16,17). Real-time polymerase chain reaction (PCR) analysis for 7 *NOS3* polymorphisms was performed using an ABIPRISM 7500 Sequence Detection System (Applied Biosystems, Foster City, CA, USA) and pre-developed TaqMan assay reagents. PCR was carried out according to the manufacturer's protocol.

### Statistical analysis

Prior to association analysis, we performed quality control of the obtained genotypes. We tested genotyping rate, Hardy-Weinberg equilibrium (HWE), and minor allele frequencies (MAF) for all samples using HaploView 4.1. MAFs were compared with the National Center for Biotechnology Information SNP database (NCBI dbSNP), MAFs for the Central European population (*www.ncbi.nlm.nih.gov/projects/SNP/*). Case-control single-point and multi-point association analyses were carried out using Haploview 4.1. Haplotype frequencies were estimated using the expectation-maximization algorithm implemented in HaploView 4.1. *P*-value lower than 0.05 was considered significant. Calculations of 80% power study at α = 0.05 were performed using Quanto. The results are expressed as OR. When OR>1, genotype confers sensitivity to the effects of exposure (18).

## Results

**Figure 1:**
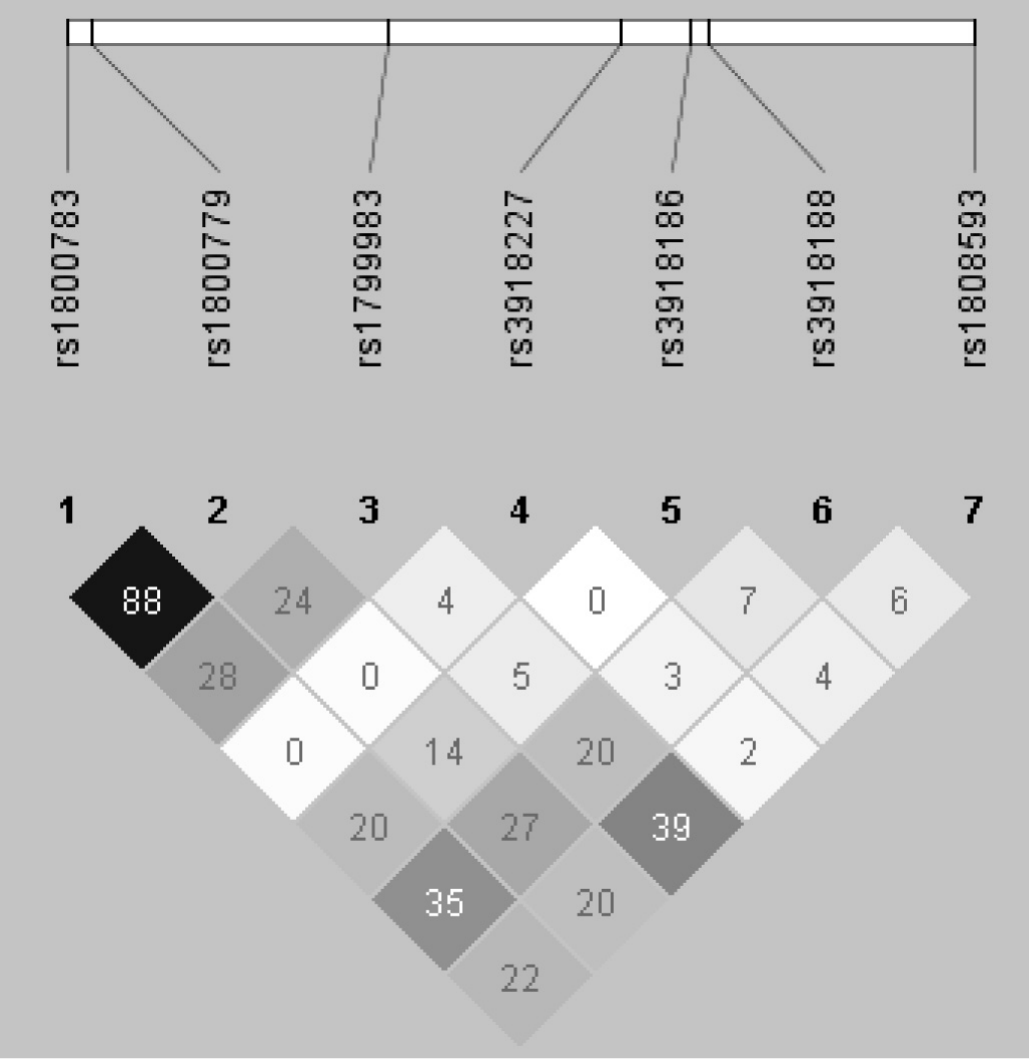
Linkage disequilibrium r^2^ values of 7 *NOS3* single nucleotide polymorphisms in the affected group of children with hypoxic-ischemic encephalopathy, calculated using HaploView.

The control group of 128 participants consisted of 38.2% of full-term infants (≥37 gestation weeks), 26.6% of preterm infants of 32-36 gestation weeks, and 35.2% of preterm infants of <32 gestation weeks, while HIE group of 110 infants consisted of 18.2% of full-term infants, 34.5% of preterm infants of 32-36 gestation weeks, and 47.3% of preterm infants of <32 gestation weeks. Genotype call rate was 100% for all the investigated SNPs. Genotypes in cases and controls fit HWE, except for rs1808593, which slightly deviated from HWE in controls (*P* = 0.0023). MAFs in controls were concordant with NCBI dbSNP frequencies for the Central European population. The 6 analyzed *NOS3* tagSNPs – rs3918186, rs3918188, rs1800783, rs1808593, rs3918227, rs1799983 – captured 100% of *NOS3* common variation at the r^2^ = 0.8, based on the HapMap phase II data. Linkage disequilibrium pattern of analyzed SNPs is shown in Figure 1. Allelic test did not find any SNP association with HIE (Table 1). However, genotypic test detected an association of rs1808593 tagSNP with HIE (χ^2^ = 9.625, *P* = 0.0081). In the group of patients with HIE, TG genotype was found less frequently and GG genotype more frequently than in controls (Table 2). We also observed rs1800783-rs1800779 TG haplotype association with HIE (χ^2^ = 11.769, *P* < 0.001) (Table 3). Genotypic and allelic distribution did not correlate with gestation age in either HIE or control group.

**Table 1:** Case-control results of 7 investigated *NOS3* single nucleotide polymorphism (SNP) in hypoxic-ischemic encephalopathy group of 110 affected children and 128 controls

SNP	Minor allele	MAF*-cases	MAF-controls	χ^2^	*P*
rs1800783	A	0.368	0.406	0.722	0.3956
rs1800779	G	0.405	0.398	0.018	0.8922
rs1799983	T	0.290	0.300	0.055	0.8141
rs3918227	A	0.081	0.082	0.0	0.9933
rs3918186	T	0.113	0.117	0.015	0.9038
rs3918188	A	0.377	0.340	0.722	0.3955
rs1808593	G	0.227	0.281	1.809	0.1787

*MAF – minor allele frequencies.

**Table 2:** Genotype analysis of 7 investigated *NOS3* single nucleotide polymorphisms (SNP) with hypoxic-ischemic encephalopathy (HIE)

	No. (%) of participants in			
SNP genotype	HIE group	control group	χ^2^	*P*
**rs1800783**				
TT	43 (39.1)	43 (33.6)	0.821	0.6633
TA	53 (48.2)	66 (51.6)		
AA	14 (12.7)	19 (14.8)		
**rs1800779**				
GG	17 (15.5)	18 (14.0)	0.063	0.968
GA	55 (50.0)	66 (51.6)		
AA	38 (34.5)	44 (34.4)		
**rs1799983**				
GG	52 (47.3)	60 (46.9)	0.28	0.869
GT	52 (47.3)	59 (46.1)		
TT	6 (5.4)	9 (7.0)		
**rs3918227**				
CC	93 (84.5)	108 (84.4)	0.0159	0.9924
CA	16 (14.5)	19 (14.8)		
AA	1 (1.0)	1 (0.8)		
**rs3918186**				
AA	87 (79.1)	101(78.9)	0.081	0.96
AT	21 (19.1)	24 (18.8)		
TT	2 (1.8)	3 (2.3)		
**rs3918188**				
CC	42 (38.2)	53 (41.4)	1.14	0.5729
CA	53 (48.2)	63 (49.2)		
AA	15 (13.6)	12 (9.4)		
**rs1808593**				
TT	67 (60.9)	59 (46.1)	9.625	0.0081
TG	36 (32.7)	66 (51.6)		
GG	7 (6.4)	3 (2.3)		

**Table 3:** Association analysis of *NOS3* rs1808783-rs1800779 haplotypes with hypoxic-ischemic encephalopathy (HIE)

Haplotype	HIE group frequencies	Control group frequencies	χ^2^	*P*
TA	0.586	0.594	0.027	0.08705
AG	0.359	0.398	0.777	0.3781
TG	0.021	0.046	0.000	<0.001

Our study had 80% statistical power to detect (α = 0.05) an effect with odds ratio (OR) = 2.07 for rs3918186, OR = 1.69 for rs3918188, OR = 1.70 for rs1800783, OR = 1.80 for rs1808593, OR = 2.10 for rs3918227, OR = 1.68 for rs1800779, and OR = 1.76 for rs1799983, assuming an additive model.

## Discussion

Our study did not demonstrate the association of any SNP with HIE. SNP rs1808593 showed genotype association and rs1800783-rs1800779 TG haplotype showed an association with HIE. Several recently published articles applied similar approach to the analysis of tagging polymorphisms but in correlation with other vascular disorders (19,20).

Nitric oxide produced in blood vessels is a well-known and important vasodilatator with a pivotal role in maintaining vascular tone in both the cerebral and systemic circulation (4,11). Hypoxia-ischemia increases the influx of calcium in endothelial cells of blood vessels and activates NOS3, which leads to generation of nitric oxide, relaxation of smooth muscle cells, regulation of the blood flow through the tissue, and reduction of harmful effects of hypoxia and ischemia (4). A number of studies have investigated the association of polymorphisms in *NOS3* gene with the occurrence of cerebral palsy, however only two studies have confirmed it (21,22). It seems that the polymorphism rs1799983 (-894G>T) located in the promotor region and the 5 tandem repeats in a 27-bp sequence in intron 4 of *NOS3,* 4a4b, are associated with variable plasma levels of nitric oxide (23,24). Also the rare C allele of the -786T>C (rs2070744) polymorphism in the 5′-flanking region of the gene has been shown to significantly reduce the activity of *NOS3* promoter (25). We observed the associations of rs1800783 (-1474 T/A) - rs1800779 (-922 G>A) haplotype and perinatal HIE. The frequency of the rare rs1800783-rs1800779 TG haplotype in our study was 2% in cases and 4% in controls, suggesting that it might have a protective role against HIE. However, since it is rare it is unlikely to have a major effect for disease development in the population. One possible explanation of the biological mechanism through which it (or a causal variant that it is tagging) may exert its function is that it influences mRNA levels of *NOS3* gene. The polymorphisms rs1800783 and rs1800779 are located in the upstream and promoter region of the gene, which may influence mRNA transcription and reduce gene expression and may further lead to impaired production or reduced bioavailability of nitric oxide, causing susceptibility to severe clinical conditions in perinatal period (11,26). In fact, beneficial effects of inhaled nitric oxide in perinatology have been observed, especially in children with respiratory distress syndrome and bronchopulmonary dysplasia (27-29). Also, the treated infants had reduced incidence of brain injury and better neurodevelopmental outcome than the control group at the age of 2 years (30-32).

So far, two studies have shown a correlation of *NOS3* -922A (rs1800779) polymorphism with cerebral palsy in preterm infants (16,17). The first study observed *NOS3* A-922G heterozygous AG and homozygous GG genotypes more frequently in the affected children. They also showed an association of functional missense *NOS3* polymorphism rs1799983 (–894 G/T or Glu298Asp) with cerebral palsy, however we did not replicate this finding in our study (16). The second study revealed the association between *NOS3* promoter -922A (rs1800779) polymorphism and preterm birth among Caucasian children with cerebral palsy, but found no correlation for the second analyzed polymorphism, rs1799983 (-894 G/T) (Glu298Asp) of the same gene (17,33). In the present study, heterozygous TG genotype of rs1808593 SNP was found less frequently, whereas homozygous GG genotype was found more frequently in the group of HIE patients than in controls. However, genotypes for this SNP slightly deviated from HWE in controls, which may be the reason for the observed association. Therefore, the observed genotypic association can be an incidental finding. Two studies have associated *NOS3* gene with hypoxic-ischemic brain damage, demonstrating the association of T-786 C and Glu298Asp polymorphisms (20,21).

Limitations of this study are a small sample size and potential population stratification due to possible relatedness among cases or controls in small populations (34). In light of this, future studies are needed to cover a larger part of Croatian population and provide the basis for further research in understanding of the influence of this gene in HIE development. They should also include other risk factors and clinical parameters.

In conclusion, despite the limited number of HIE patients, which reduced the statistical power of this study, we observed genotype and haplotype associations of *NOS3* polymorphisms with HIE, obtaining evidence of the important role of this gene in susceptibility to hypoxic-ischemic perinatal damage. A complete comprehension of *NOS3* genetic contribution to HIE development is vital for understanding the disease etiology and future treatment of the disease. New diagnostic, prevention, and therapeutic approaches might derive from this knowledge; for example, it might be useful to select children with high specific genetic risk for HIE development because low dose nitric oxide inhalation therapy may be a beneficial treatment. Today, such treatment is applied only in respiratory disorders (27,28) but it can also be used in the prevention hypoxic-ischemic brain damage.
